# Imagery and Verbal Strategies in Spatial Memory for Route and Survey Descriptions

**DOI:** 10.3390/brainsci14040403

**Published:** 2024-04-20

**Authors:** Ioanna Markostamou, Sol Morrissey, Michael Hornberger

**Affiliations:** 1 Department of Psychology, Bournemouth University, Poole BH12 5BB, UK; 2 Division of Psychology, School of Life and Medical Sciences, University of Hertfordshire, Hatfield AL10 9AB, UK; 3 Norwich Medical School, University of East Anglia, Norwich NR4 7UQ, UK

**Keywords:** memory strategies, imagery, route, survey, spatial memory, spatial descriptions, spatial perspective, navigation

## Abstract

Being able to represent and remember verbally-encoded information about spatial environments from different perspectives is important for numerous daily activities. The present study examined how frequently individuals spontaneously engage in visual mental imagery and verbal rehearsal strategies in memory recall of spatial descriptions, and whether using certain strategies is associated with better recall performance. Memory recall for route (person-centred) and survey (object-centred) spatial descriptions was examined in a sample of 105 neurotypical individuals, who also reported to what extent they used route- and survey-based mental imagery and verbal rehearsal strategies in each description. Results showed that participants favoured a path visualisation strategy to recall the route description and a map visualisation strategy to recall the survey description. Regression models further showed that employing both imagery and verbal strategies was associated with better recall for both route and survey descriptions, although imagery strategies held a higher predictive power. The present findings highlight the fact that the spontaneous use of internal strategies in the form of visual imagery and verbal rehearsal is ubiquitous when recalling spatial descriptions and a core part of efficient spatial memory functioning.

## 1. Introduction

Mental representations of spatial information typically develop through direct sensorimotor experience in the environment. However, spatial mental representations and spatial knowledge can also derive verbally from spatial descriptions [1,2,3,4,5]. The close relation between verbal and non-linguistic representations of space has been documented across behavioural [6,7], psychometric [8], neuropsychological [9,10], developmental [8,11], and neuroimaging [12,13] investigations. For example, verbally describing spatial relations between objects has been found to rely heavily on visuospatial abilities [8] and to recruit parietal neural regions which are typically associated with visuospatial operations [12]. Such findings support substantial overlaps in the mental and neural operations that support linguistic and perceptual representations of space.

Being able to spatially represent and remember verbally-encoded information about spatial environments is important for numerous daily and professional activities, such as giving or following wayfinding and navigational directions. It is well established that people can form spatial mental representations from spatial descriptions that accurately maintain the spatial properties and relationships between the elements of the described environment [2,4,14]. For example, existing evidence indicates that spatial representations derived from visuoperceptual encoding or from spatial descriptions are equivalent in supporting the development of spatial relational knowledge [15]. However, it is not clear to what extent individuals rely on verbal or visuospatial processes, or both, while encoding such information. Moreover, it is not clear whether the spatial perspective in which the information is presented influences spontaneous strategy choice, and which strategies are most beneficial for memory recall. The present study aims to address these questions.

Spatial representations can develop within different reference frames and perspectives [16,17,18]. Egocentric spatial representations are encoded and updated relatively to the changing perspective of an observer whereas allocentric spatial representations involve spatial relations that are centred on an external reference object, independent of the perceiver’s position or orientation [19,20]. In the case of spatial descriptions, route descriptions are based on a person-centred (or egocentric) perspective, with spatial relations being defined by the changing viewpoint of an agent (e.g., “Zoe is in front of the Library”). Spatial relations in survey descriptions are based on a stable object-centred (or allocentric) perspective, and are independent from the changing viewpoint of the perceiver (e.g., “The Library is opposite the Forum”) [3,5].

Representing and recalling spatial information from spatial descriptions can be subject to large individual differences in both verbal and visuospatial abilities, such as verbal and visuospatial working memory resources [2,3,21,22]. Previous investigations have often used dual task paradigms, in which participants performed secondary tasks taxing either their visuospatial (e.g., spatial tapping) or verbal (e.g., articulatory task) working memory resources while hearing or reading spatial descriptions. Experimental evidence from those studies has shown that both verbal and visuospatial working memory capacity is important in maintaining and recalling route descriptions [21,22] while visuospatial working memory seems pivotal for retaining spatial information from survey descriptions [2,23]. Studies employing an individual differences approach have correspondingly supported the potential involvement of verbal and visuospatial resources in recalling spatial descriptions, which nevertheless vary depending on the perspective involved. Specifically, while both verbal and visuospatial working memory capacity have been linked to route recall, only visuospatial working memory has been associated with memory recall of a survey description [3].

Another source of intra- and inter-individual variation in memory performance can be individual differences in employing memory strategies [24]. Memory strategies refer to any helpful technique used to enhance the encoding and retrieval of information, ranging from deliberate use of external aids and cognitive offloading techniques (e.g., writing notes or setting an alarm [25] to the spontaneous (i.e., self-initiated) generation of internal strategies like verbal rehearsal and mental imagery [26,27]. Rehearsal has traditionally been conceptualised as a memory strategy for maintaining information over time through the (usually silent) repetition and processing of the propositional or phonological contents of the memory to oneself. By contrast, using visual mental imagery as a mnemonic strategy to retain information involves the creation of mental images picturing the memory contents in one’s mind.

Identifying which types of strategies people use to perform different spatial memory and navigation tasks is particularly important in understanding the processes underpinning efficient spatial learning and memory [28,29,30,31,32,33,34,35,36]. A key aim of the present study is to examine the use of language- and imagery-based strategies that individuals employ to remember verbally encoded spatial information presented from a route and a survey perspective. More specifically, we examine to what extent individuals spontaneously engage in mentally rehearsing and processing the propositional information of the spatial descriptions or in mentally constructing a visuospatial representation of the spatial information described.

Individuals typically exhibit a preference in processing information either by imagery or verbal processes and strategies, reflecting a visualizer vs. a verbalizer cognitive style [27,37]. Given the close connection between linguistic and non-linguistic representations of space [8], and the contribution of both verbal and visuospatial cognitive resources in forming, maintaining, and retrieving spatial information from spatial descriptions [2,3,4,14], we expected that both verbal- and imagery-based strategies would be spontaneously employed by participants. However, visuospatial resources appear to have a more prominent role in supporting memory recall of spatial descriptions [2,3,23] and one’s processing approach and strategy use can flexibly change depending on the given task [27]. In fact, previous empirical evidence suggests that task characteristics exert a greater influence than individual preferences towards using verbal or visual strategies when encoding navigational spatial information [32]. In addition, instructing participants to use imagery strategies has been found to be more beneficial than verbal strategies in constructing and maintaining spatial mental representations of route descriptions [14,38]. It is therefore likely that participants will be less reliant on verbal processing and instead spontaneously choose mental imagery as a more efficient strategy to generate and retain internal representations of the environmental descriptions.

Another key aim of the present study is to examine the types of imagery strategies individuals employ to remember spatial descriptions, and whether using certain imagery strategies—particularly a path (i.e., route) visualisation strategy or a map (i.e., survey) visualisation strategy—is associated with better memory recall depending on the perspective involved in the spatial description (i.e., route and survey). As in spatial navigation learning and memory paradigms [16,29,31,34,39,40,41], route-based strategies (also called response learning strategies) involve representing the sequence of places and landmarks and the turns along a route from the perspective of the navigator (i.e., egocentrically). By contrast, survey-based strategies (also called place learning strategies) involve representing the layout of an environment in the form of a cognitive map that features allocentric object-to-object spatial relations [16,29,31,34,39,40,41].

Egocentric and allocentric representations can be formed simultaneously when learning a new environment; however, the prevalence of one perspective over the other varies, depending on task characteristics [42,43]. Furthermore, the formation of spatial mental representations appears to be orientation-dependent and aligned with the spatial reference frame established during encoding (for example, see [40,41,44,45]). Therefore, while it is possible that participants’ mental representations will incorporate both path- and survey-based imagery for both spatial descriptions, we expected that they would spontaneously adapt their choice of imagery strategy so that it is aligned with the reference frame provided in the description to optimally meet the spatial task demands. We also expected that flexibly switching and employing a congruent imagery strategy for each respective perspective at encoding (i.e., developing path-based mental imagery during the route description and map visualisation during the survey description) would be associated with higher spatial memory recall at the test phase.

To summarise, the aims of the present study are threefold: (a) to examine to what extent individuals spontaneously engage in verbal and visuospatial (path visualisation and map visualization) strategies when developing mental representations from spatial descriptions, (b) to determine whether strategy choice depends on the perspective involved (route, survey), and (c) to identify which strategies are associated with better memory recall of spatial frame-specific environmental descriptions.

## 2. Materials and Methods

### 2.1. Participants

A total of 105 participants (58% female) were recruited for this study. Participants were between 18 and 43 years of age (*M* = 21.08, *SD* = 3.99) and had completed on average 13–14 years of formal schooling (*M* = 13.68, *SD* = 2.08).

Most participants were university students who were recruited through an online research participation system and received course credits for their participation. The remaining participants were recruited from the local community through invitation leaflets and word of mouth.

All participants were fluent in English and had normal (or corrected-to-normal) vision and hearing. Participants had no active neurological or psychiatric condition affecting cognitive functioning, nor prior history of head injury or substance dependence. The initial sample included three more participants (*N* = 108) who were excluded because of missing data (one participant) or having had an active neuropsychiatric condition (two participants).

### 2.2. Materials and Measures

#### 2.2.1. Spatial Descriptions

We used the Spatial–Verbal Memory test (SVM) to assess memory recall for route and survey spatial descriptions [3]. The SVM consists of two spatial descriptions, which contain spatial information presented from a person-centred perspective (route description) and an object-centred perspective (survey description), respectively. Each description contains 25 semantic units, 10 of which provide spatial information. In the route description, locations of landmarks are described relative to the perspective of a protagonist walking on a mountain path (e.g., “He kept the lake on his right, until he passed under a large oak tree”). In the survey description, locations of landmarks in a town centre are described from an object-centred perspective (e.g., “The library is situated in front of the church and to the right of the Town Hall”; for more information, see [3]).

Participants were informed that they would hear the short stories and they should try to remember each one as closely to the original as possible, in order to be able to recall them later. Participants heard each spatial description two times (encoding phase) and were asked to verbally recall it immediately after hearing it (immediate recall trial) as well as after a 25 min delay (delayed recall trial). Participants’ responses were recorded separately during the immediate and delayed recall trials, and each correctly recalled unit was scored one point (maximum score: 25). In addition, each correctly recalled spatial information unit was separately identified and scored one point in each recall trial of each description (maximum score: 10).

#### 2.2.2. Memory Strategies

Unless participants are specifically instructed to use certain strategies (e.g., [38,46]), constructs regarding memory strategy use are often examined by self-report measures (e.g., [4]). Participants were asked to indicate to what extent they had used different types of memory strategies for each spatial description during the encoding stage after each delayed recall trial, using a four-item questionnaire.

For the route description, the first item described a verbal rehearsal strategy (“I processed and repeated the verbal content mentally”). The second item described a route-based mental imagery strategy (i.e., a path visualisation strategy; “I imagined moving along the path/I took the path mentally”). The third item described a survey-based mental imagery strategy (i.e., a map visualisation strategy; “I created a mental map/I built a map mentally”). A fourth item was used for any other memory strategy employed (“I used another memory strategy”), and, if used, participants described it in their own words.

For the survey description, the first item was identical to the first item used in the route description and referred to a verbal rehearsal strategy (“I processed and repeated the verbal content mentally”). The second item described a route-based mental imagery strategy (i.e., a path visualisation strategy; “I imagined moving along the landmarks/I created a path mentally”). The third item referred to a survey-based mental imagery strategy (i.e., a map visualisation strategy; “I created a mental map/I built a map mentally”). A fourth item was again used for any other memory strategy employed, which participants described in their own words.

Participants reported the extent to which they had used each type of strategy for each spatial description on a monopolar 5-point scale (1 = not at all; 2 = to little extent; 3 = to some extent; 4 = to a large extent; 5 = to a great extent). Each question along with the corresponding response options were read by the experimenter while at the same time they were also presented to the participants in a printed format on A5-sized cards. Participant strategy use ratings (ranging from 1 to 5) for each type of strategy (i.e., verbal rehearsal, path visualisation, map visualisation) for each spatial description (i.e., route, survey) were used as the dependent variables.

### 2.3. General Procedure

Ethics approval was obtained from the local ethics committee and all study procedures were carried out in accordance with the British Psychological Society guidelines and the 2013 Declaration of Helsinki. All participants took part voluntarily and provided written informed consent.

Participants were tested in a single individual lab session lasting between 45 and 60 min, that took place at the university campus. At the outset of the testing session, each participant provided demographic information and then completed the encoding and immediate recall trials of the route and survey descriptions in a random order. The delayed recall trials took place approximately 25 min after the immediate recall trials and participants completed filler tasks during the interval.

Participants’ responses in each recall trial were audio recorded and later transcribed for scoring. The delayed recall trial of each description was followed by the strategy questionnaire. The rationale behind asking participants to report their strategy use after each delayed recall trial rather than at the encoding stage was to ensure that each choice of strategy use was self-generated rather than suggested to participants. Furthermore, this approach allowed us to examine whether participants spontaneously switched strategies across the route and survey perspectives.

### 2.4. Data Analysis

Data analysis is presented in three main sections. In the first section, we examine memory recall performance with a series of paired-samples *t*-tests.

The second section focuses on memory strategy use. A 2 (perspective: route and survey) by 3 (strategy type: verbal rehearsal, path visualisation, map visualisation) within-subjects repeated measures analysis of variance was employed to examine strategy use. Follow up tests were conducted wherever appropriate.

In the third section, we present a series of multiple linear regressions that were conducted to determine which verbal- and imagery-based memory strategies contribute to better memory recall for route and survey descriptions.

## 3. Results

### 3.1. Memory Recall Performance

Descriptive statistics for memory recall accuracy (proportion of correctly recalled units) for the route and survey descriptions are provided in Table 1.

Overall, memory recall was substantially higher in the immediate recall trials compared to the delayed recall trials (memory recall of route description: t(104) = 9.13, *p* < 0.001, Cohen’s d = 0.89; recall of spatial information units in the route description: t(104) = 6.61, *p* < 0.001, Cohen’s d = 0.64; memory recall of survey description: t(104) = 10.01, *p* < 0.001, Cohen’s d = 0.97; and recall of spatial information units in the survey description: t(104) = 6.69, *p* < 0.001, Cohen’s d = 0.65).

In addition, higher recall rates were observed for the route description compared to the survey description (immediate recall: t(104) = 4.61, *p* < 0.001, Cohen’s d = 0.45; delayed recall: t(104) = 5.73, *p* < 0.001, Cohen’s d = 0.56). However, there were no significant differences in recalling the spatial information presented in the route and survey descriptions (immediate recall: t(104) = −0.31, *p* = 0.759, Cohen’s d = −0.03; delayed recall: t(104) = 0.44, *p* = 0.662, Cohen’s d = −0.04).

### 3.2. Strategy Use

A small minority of the participants (less than 5%) indicated they had used some strategy other than verbal rehearsal, path visualisation, and map visualisation. Of those few reports, one was a verbal-based strategy where the participant reported using phonemic cues in a particular way (i.e., “trying to remember the first letter of each landmark and create an acronym out of them”). Furthermore, two participants indicated they had engaged in a particular imagery strategy by making associations and linking the information of the spatial descriptions to prior knowledge of familiar environments (i.e., “picturing my home town and trying to change the landmarks and their locations accordingly”). Given their scarcity, those reported strategies were not retained in subsequent statistical analysis.

**Figure 1:**
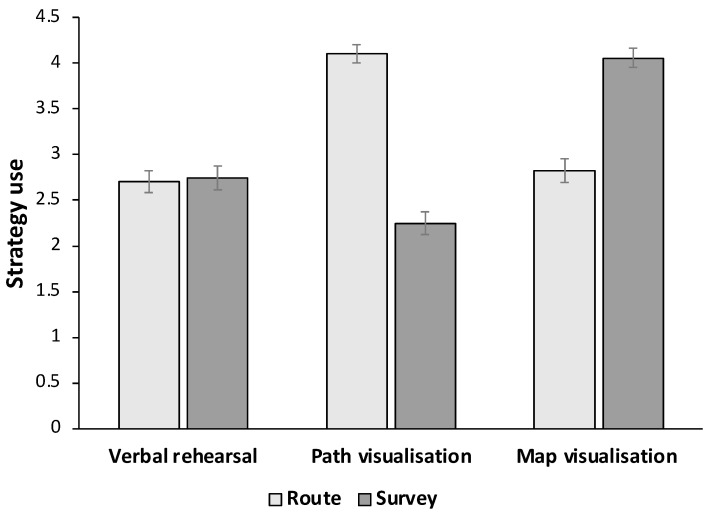
Use of verbal rehearsal, path visualisation and map visualisation in memory recall of route and survey descriptions. *Note.* Error bars show the standard error of the mean; *N* = 105.

**Figure 2:**
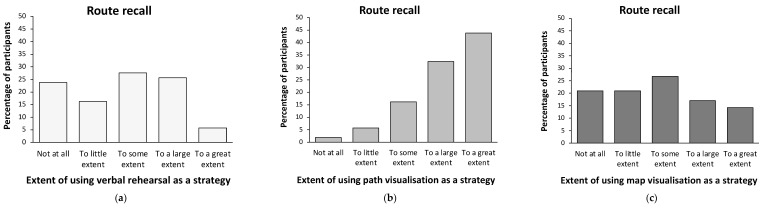
Distribution graphs of using verbal rehearsal (**a**), path visualisation (**b**), and map visualisation (**c**) memory strategies in the route description among participants.

**Figure 3:**
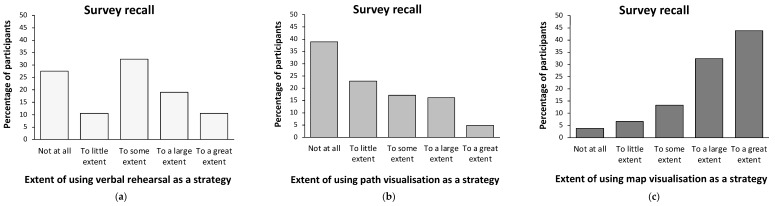
Distribution graphs of using verbal rehearsal (**a**), path visualisation (**b**), and map visualisation (**c**) memory strategies in the survey description among participants.

Figure 1 presents the average verbal-rehearsal, path-visualisation, and map-visualisation strategy use for the route and survey descriptions across all participants. The distribution of using each verbal and imagery strategy across the route and survey descriptions are presented in Figure 2 and Figure 3, respectively.

Results of a 2 (perspective: route and survey) by 3 (strategy type: verbal rehearsal, path visualisation, map visualisation) repeated-measures analysis of variance yielded a medium effect of perspective on strategy use, F(1, 104) = 8.66, *p* = 0.004, ηp^2^ = 0.08. Overall, participants reported using memory strategies to a greater extent for the route description (M = 3.21, SD = 1.20) compared to the survey description (M = 3.02, SD = 1.22).

A large effect of strategy type on strategy use was also observed, F(2, 208) = 17.17, *p* < 0.001, ηp^2^ = 0.14. Pairwise comparisons revealed that, overall, map visualisation was used to a greater extent than verbal rehearsal (*p* < 0.001) and path visualisation (*p* = 0.026). Moreover, path visualisation was used to a greater extent than verbal rehearsal (*p* = 0.005) (verbal rehearsal: M = 2.72, SD = 1.30; path visualisation: M = 3.18, SD = 1.13; map visualisation: M = 3.44, SD = 1.21).

Importantly, those effects were qualified by a large perspective by strategy type interaction effect on strategy use, F(2, 208) = 93.99, *p* < 0.001, ηp^2^ = 0.48. A set of post hoc pairwise comparisons revealed that while there was no difference in the extent of using verbal rehearsal across the route and survey descriptions, t(104) = −0.31, *p* > 0.250, Cohen’s d = −0.03, using path visualisation was higher in the route description compared to the survey description, t(104) = 12.25, *p* < 0.001, Cohen’s d = 1.19, and using map visualisation was higher in the survey description compared to the route description, t(104) = −7.48, *p* < 0.001, Cohen’s d = −0.73 (Figure 1).

In the route description, post hoc pairwise comparisons showed that path visualisation was significantly higher than both verbal rehearsal, t(104) = 8.66, *p* < 0.001, Cohen’s d = 0.85, and map visualisation, t(104) = 7.84, *p* < 0.001, Cohen’s d = 0.76, while there was no significant difference between verbal rehearsal and map visualisation, t(104) = −0.77, *p* = 0.221, Cohen’s d = −0.07 (Figure 1). Of note, less than 2% of the participants reported they had not used a path visualisation strategy at all to mentally represent and retain the route description, whilst approximately 21% to 25% of the participants reported they had not used map visualisation and verbal rehearsal for the route description, respectively (Figure 2). In addition, more than three quarters of the participants (76.2%) reported using a path visualisation strategy to a large or to a great extent, while, in contrast, about a third of the participants (31%) reported employing a verbal rehearsal strategy or a map visualisation strategy to a large or to a great extent for the route description (Figure 2).

In the survey description, post hoc pairwise comparisons showed greater use of map visualisation over verbal rehearsal, t(104) = 7.62, *p* < 0.001, Cohen’s d = 0.74, and path visualisation, t(104) = 10.77, *p* < 0.001, Cohen’s d = 1.05 (Figure 1). Furthermore, using verbal rehearsal was greater than path visualisation, t(104) = −2.71, *p* = 0.008, Cohen’s d = −0.26 (Figure 1). More specifically, less than 4% of the participants reported not having used a map visualisation strategy at all while encoding the survey description, while more than 38% of the participants reported they had not used a path visualisation strategy and about 28% had not used a verbal rehearsal strategy (Figure 3). On the other hand, while more than three quarters of the participants (76%) reported relying on a map visualisation strategy to a large or to a great extent, about one out of five participants (21%) reported using a path visualisation strategy to a large or to a great extent. About a third of the participants reported they had employed a verbal rehearsal strategy to a large or to a great extent in order to enhance their memory of the survey description (Figure 3).

### 3.3. Regression Models

A series of multiple linear regressions were conducted to determine which verbal- and imagery-based memory strategies contribute to better memory recall for route and survey descriptions. Given that immediate recall is related more to short-term and working memory capacity than to long-term memory processes involved in recall after a delay, performance in the delayed recall trials was used for this analysis [3]. The results of the regression models are presented in Table 2.

#### 3.3.1. Route Description

**Figure 4:**
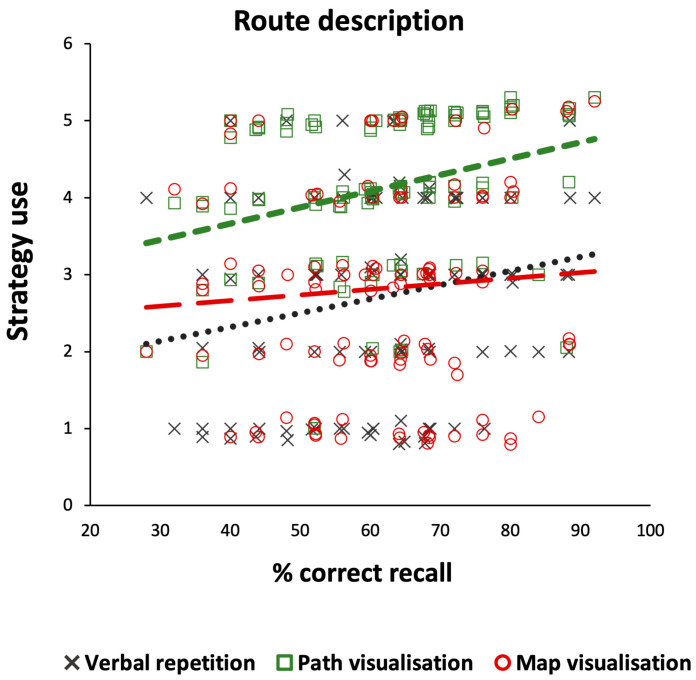
Scattergram of memory recall performance as a function of the type of strategy used (verbal repetition, path visualization, map visualization) for the route description. *Note.* For demonstration purposes, data points have been spaced by a 0.01–0.09% margin to reduce their visual overlapping; *N* = 105.

The regression model showed that both verbal rehearsal and path visualisation strategy use was associated with memory recall performance for the route description, F(3, 101) = 3.45, R = 0.32, *p* = 0.019, and accounted for 9% of the variance. Path visualisation held the highest predictive power for the route recall (Table 2; see also Figure 4).

A separate regression model revealed that only path visualisation strategy use contributed to memory recall for the spatial information contained in the route description, F(3, 101) = 3.24, R = 0.30, *p* = 0.025, and accounted for 9% of the variance (Table 2).

#### 3.3.2. Survey Description

**Figure 5:**
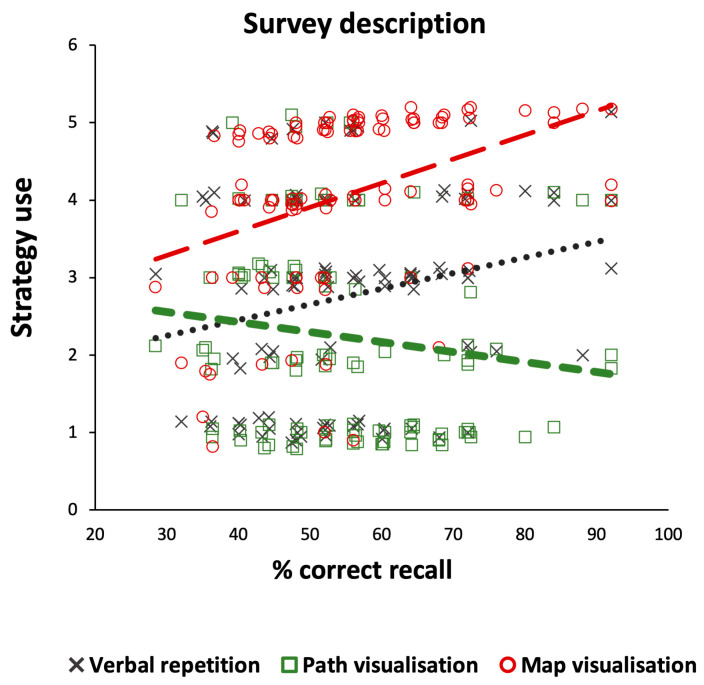
Scattergram of memory recall performance as a function of the type of strategy used (verbal repetition, path visualization, map visualization) for the survey description. *Note.* For demonstration purposes, data points have been spaced by a 0.01–0.09% margin to reduce their visual overlapping; *N* = 105.

The results of the regression analysis showed that both verbal rehearsal and map visualisation strategies were significant predictors of memory recall performance for the survey description, F(3, 101) = 6.01, R = 0.39, *p* < 0.001, and accounted for 15% of the variance. Map visualisation was found to hold the highest predictive power for the survey recall (Table 2; see also Figure 5).

A similar regression model also showed that both verbal rehearsal and map visualisation strategies contributed to memory recall for the spatial information of the survey description, F(3, 101) = 2.89, R = 0.28, *p* = 0.039, and accounted for 8% of the variance. The standardized coefficients showed that verbal rehearsal and map visualisation held a similar predictive power for the survey recall (Table 2).

## 4. Discussion

In the present study, we examined the extent of spontaneous, self-initiated (i.e., uninstructed) use of visual mental imagery and verbal rehearsal strategies in memory of spatial descriptions. We also examined whether strategy choice varies depending on the perspective provided in the spatial description, including a person-centred route description and an object-centred survey description. Furthermore, we examined which verbal and imagery strategies are associated with better memory recall of route and survey descriptions. To address these questions, a cohort of young neurotypical adults completed a spatial–verbal memory test [3], which involves listening to route and survey spatial descriptions and then freely recalling them. Participants also reported to what extent they had engaged in mentally rehearsing and processing the propositional and/or phonological information of the spatial descriptions (verbal rehearsal strategy) or in mentally constructing path-based or map-based visuospatial representations of the description (path visualisation and map visualisation mental imagery strategies).

Several important findings emerged from this study that shed light on the underlying representational operations taking place when encoding and processing descriptions of spatial environments with the intention to retain them for subsequent recall. First, we found that the self-initiated use of both verbal- and imagery-based strategies is highly prevalent in spatial memory tasks involving verbally encoded information. More specifically, irrespectively of the perspective involved (i.e., route, survey), about a third of the study’s participants reported using a verbal strategy to a large or to a great extent while encoding the spatial descriptions. Furthermore, about a third of the participants had relied at least to some extent on verbal rehearsal, while a third of the participants indicated they had not employed verbal rehearsal at all or to a limited extent. Meanwhile, the majority of the participants (around three quarters of the participants) indicated they had used mental imagery strategies to a large or to a great extent while forming mental representations derived from the spatial descriptions. Moreover, higher strategy use of both verbal- and imagery-based strategies during encoding was associated with higher memory retrieval at the test phase.

These findings highlight that the spontaneous use of internal strategies in the form of visual imagery and verbal rehearsal is ubiquitous when encoding and developing spatial mental models from route and survey descriptions and a core part of efficient spatial memory functioning. From a theoretical standpoint, these findings are consistent with the notion of a flexible supramodal cognitive system supporting spatial representations within the verbal and visuoperceptual domains [8,15,47]. Modal strategy use along the verbal and visuospatial domains in learning, memory, and navigation tasks often depends on individual preferences as well as specific task characteristics [4,32], although more than one strategy can be employed [24,27]. The concurrent reliance on both verbal and imagery strategies observed in the present study suggests that individuals spontaneously recruit any cognitive resources and tools across the verbal and visuospatial domains that are available to them while they form spatial representations from navigational descriptions. At the same time, this finding confirms that verbal and visuospatial strategies may be operating synergistically to support efficient encoding and processing of spatial descriptions and can ultimately enhance memory performance. Comparable findings of a beneficial effect of using verbal and spatial strategies have been reported in investigations of route learning through visual encoding [46]. Similarly, visual mental imagery has been found to be a strong predictor of intentional [48] as well as incidental recall of verbal material [49]. In fact, several seminal theoretical accounts have emphasized the importance of the interaction between language and visuoperceptual simulation operations on memory and cognition, placing the multimodal representation and processing of information at the heart of efficient cognitive and memory functioning [27,36,50,51].

However, employing imagery strategies was found to play a more salient role than verbal strategy use in recalling route and survey descriptions. These findings corroborate and extend previous evidence of the beneficial role of visual imagery use in memory recall of route descriptions [14,38]. In fact, there is an increasing number of empirical evidence suggesting that visual mental imagery plays a vital role across a wide range of learning and memory phenomena, including spatial memory and navigation [52,53], verbal learning and memory [48,49], recalling past autobiographical events [54], prospective memory [55], and episodic future thinking [56].

A second set of important findings concerns the variability observed in the extent of using different types of imagery strategies depending on the perspective involved. Interestingly, participants reported employing, at least to some extent, both path-based and map-based imagery across both spatial descriptions. This finding accords with the notion that mentally representing spatial relations from different perspectives can occur automatically during early encoding stages [57] and may act complementary to forming comprehensive spatial mental models [19,39]. For example, studies employing navigation paradigms have shown that learning the sequence of places encountered along a route of a virtual environment supports the formation of cognitive maps of the environment [58].

Nevertheless, the degree to which path-based and map-based imagery was used varied according to the perspective provided in the spatial description, with participants clearly favouring a path visualisation imagery strategy while encoding the route description and a map visualisation imagery strategy to recall the survey description. Thus, instead of employing a fixed (putatively preferred) approach of mentally processing spatial information either egocentrically or allocentrically, the perspective-dependent variation in mental imagery use provides evidence of a flexible adaptation in the development of spatial mental representations along the egocentric and allocentric frames of references depending on the perspective in which the information was presented. Moreover, using a visual mental imagery strategy during encoding that maintained the same perspective in which the spatial relations were presented in each description was associated with higher memory recall at test. Overall, these findings point to a flexible switch in using mental imagery within different perspectives that enables the described spatial information and their corresponding spatial mental representations to be aligned, and ultimately promotes optimum encoding and memory retention of the information. These observations echo previous empirical reports suggesting that the development of spatial mental representations tends to be perspective-dependent and aligned with the given orientation established during encoding [40,41,44,45]. Furthermore, our findings are well aligned with previous reports of a flexible mental switch between the egocentric and allocentric strategies individuals employ to perform different tasks in virtual navigation paradigms [29] and that a self-initiated, task-dependent switch between egocentric and allocentric strategy use is crucial for efficient spatial memory and navigation [34].

Examining memory strategy use for spatial descriptions in special populations like older adults or individuals with cognitive/spatial deficits was beyond the scope of the present study. However, the practical implications of the present findings extend to applied and clinical settings for individuals with potential impairments in spatial memory and navigation, including older adults—both healthy and with neurodegenerative conditions [3,33,34,59,60]. If poorer navigational and spatial memory performance among typically and atypically ageing individuals is at least partially mediated by sub-optimal strategic processing (e.g., [33,34]), future research should examine whether prior knowledge and training on efficient strategy use may ameliorate spatial navigation and memory difficulties. The beneficial role of verbal and imagery memory strategies can be further examined in neuropsychological rehabilitation investigations (e.g., [16,61]).

## 5. Conclusions

The findings of the present study provide important insights about the representational operations taking place when encoding and processing route and survey spatial descriptions. First, we found that the self-initiated use of internal verbal- and imagery-based strategies is highly prevalent in spatial memory tasks involving verbally encoded spatial information. Second, the degree to which both verbal as well as imagery strategies were used contributed to memory recall performance, underscoring the importance of memory strategy use in efficient learning and memory functioning and highlighting the significance of multimodal processing of spatial information in memory retention and recall. Third, we observed a spontaneous shift in the path and map imagery strategy selection in a way that ensured that the development of spatial representations maintained the spatial perspective involved in the description, confirming that the strategic processing of navigational information is flexible, depending on the spatial perspective, and driven by task characteristics. Taken together, the results of the present study are indicative of a flexible morphosis of verbal as well as perspective-dependent spatial representations along the egocentric and allocentric frames of reference when one is encoding and processing route and survey spatial descriptions.

## Figures and Tables

**Table 1:** Descriptive statistics for all memory recall trials for the route and survey descriptions.

	*Mean*	*SD*	*Min*	*Max*	Distribution	
					*Skewness*	*Kurtosis*
**Route description**						
Immediate route recall	68.72	13.80	40.00	96.00	−0.200	−0.636
Immediate recall of spatial information	57.14	19.79	10.00	100.00	−0.170	−0.610
Delayed route recall	61.68	14.08	28.00	92.00	−0.167	−0.285
Delayed recall of spatial information	49.81	17.12	10.00	90.00	−0.254	−0.328
**Survey description**						
Immediate survey recall	63.80	13.84	32.00	96.00	−0.021	−0.418
Immediate recall of spatial information	57.62	18.47	20.00	90.00	−0.118	−0.876
Delayed survey recall	55.20	13.92	28.00	92.00	0.686	0.172
Delayed recall of spatial information	49.05	16.10	20.00	90.00	0.456	−0.283

*Note*. Mean (SD) values represent percentages of correctly recalled units; *N* = 105.

**Table 2:** Regression summaries for the route and survey memory recall as predicted by verbal-rehearsal, path-visualisation, and map-visualisation memory strategy use.

Predictors	*F* (3, 101)	*R*	*R* ^2^	*B* (*SE*)	*β*	*t*-Value
**Route description**						
*Route recall*	3.45 **	0.32	0.09			
Verbal rehearsal				0.55 (0.27)	**0.20**	20.05 *
Path visualisation				0.84 (0.33)	**0.24**	20.51 **
Map visualisation				0.04 (0.25)	0.02	00.17
*Recall of spatial information*	3.24 *	0.30	0.09			
Verbal rehearsal				0.14 (0.13)	0.10	10.05
Path visualisation				0.49 (0.17)	**0.28**	10.95 **
Map visualisation				0.03 (0.13)	0.02	00.24
**Survey description**						
*Survey recall*	6.01 ***	0.39	0.15			
Verbal rehearsal				0.55 (0.24)	**0.21**	20.28 *
Path visualisation				−0.29 (0.25)	−0.10	−10.15
Map visualisation				0.99 (0.29)	**0.31**	30.39 ***
*Recall of spatial information*	2.89 *	0.28	0.08			
Verbal rehearsal				0.25 (0.12)	**0.20**	20.10 *
Path visualisation				−0.02 (0.12)	−0.02	−00.18
Map visualisation				0.31 (0.14)	**0.21**	20.15 **

*Note*. Variables with the strongest predictive power are in bold; *N* = 105; * *p* < 0.05, ** *p* < 0.01, *** *p* < 0.001.

## Data Availability

The data presented in this study are available on request from the corresponding author. The data are not publicly available due to privacy and ethical restrictions.
